# Next Steps: Studying Diabetic Foot Infections with Next-Generation Molecular Assays

**DOI:** 10.1007/s11908-023-00822-8

**Published:** 2023-10-27

**Authors:** Caitlin Sande, Zoë J. Boston, Lindsay R. Kalan, Meghan B. Brennan

**Affiliations:** 1 Department of Biochemistry and Biomedical Sciences, Health Sciences Centre, McMaster University, Room 4H41, 1200 Main St West, Hamilton, ON L8N 3Z5, Canada; 2 Department of Medicine, School of Medicine and Public Health, University of Wisconsin, 1685 Highland Ave, Madison, WI 53583, USA

**Keywords:** Microbiome, Ulcer, Amputation, Metatranscriptomics, Metagenomics, Amplicon sequencing

## Abstract

**Purpose of Review:**

In 2019, the International Working Group on the Diabetic Foot voiced six concerns regarding the use of molecular microbiology techniques for routine diagnosis of infection complicating diabetic foot ulcers. The purpose of this review is to evaluate contemporary evidence addressing each of these concerns and describe promising avenues for continued development of molecular microbiology assays.

**Recent Findings:**

Since 2019, the feasibility of conducting metagenomic and metatranscriptomic studies on diabetic foot ulcer samples has been shown. However, these preliminary studies used small samples with concerns for selection bias. We await larger-scale, longitudinal studies, potentially using the recently formed Diabetic Foot Consortium, to identify microbiome profiles associated with infection and patient outcomes. How these results would translate into a clinical diagnostic requires further clarification.

**Summary:**

High-throughput molecular microbiology techniques are not yet ready for clinical adoption as first-line diagnostics. However, moving from amplicon sequencing to metagenomic and metatranscriptomic studies has the potential to significantly accelerate development of assays that might meaningfully impact patient care.

## Introduction

Roughly 150,000 Americans undergo major, above-ankle, amputation each year [1]. Limb loss is most commonly triggered by the triad of diabetes, peripheral vascular disease, and infection [2]. The diabetes epidemic shows no signs of stopping [3]. The advent of endovascular revascularization partially reversed amputation trends in the 1990s and 2000s [4]. However, these gains could not be sustained; rates have been rising since 2009 [5]. Advances in infectious disease are now needed to meet the goal of reducing amputations by 20% over the next decade [1].

In 2019, the International Working Group on the Diabetic Foot updated guidelines on the diagnosis and treatment of infected diabetic foot ulcers [6•]. The guideline recommended against molecular microbiology techniques for first-line identification of pathogens based on six valid concerns. First, studies published at that time enrolled relatively few patients and were at risk of selection bias. Second, these studies could not distinguish between colonization and infection. Third, they could not discern living from dead organisms. Fourth, most molecular assays contained limited information about the antimicrobial susceptibilities of identified organisms. Fifth, it was unclear whether the number of organisms in a wound or the presence of specific virulence genes correlated with patient outcomes. Given these scientific limitations, molecular microbiology techniques did not clearly advance clinical prognosis or therapeutics beyond standard culture. Finally, the cost of molecular assays seemed exorbitant for the amount of information potentially gained.

We review each of these six concerns in light of published and ongoing efforts to address them. We agree that molecular microbiology techniques are not ready for guideline endorsement as of yet. We also think that collaboration between clinicians and molecular microbiologists is key to developing molecular assays that meet medical needs. Finally, outside of direct applications, the complexity of wound microbiomes is, quite simply, fascinating. Understanding how biodiverse microbial communities assemble in wound tissue and shift from harmony to haywire infection is a story that is sure to captivate both clinical and basic microbiologic audiences.

Before addressing each of the six concerns point-by-point, we offer a brief review of three molecular techniques highlighted below: amplicon sequencing, metagenomics, and metatranscriptomics (Table 1). Amplicon sequencing, also known as 16S amplicon sequencing, is the most mature of these three methods. It uses high-throughput sequencing technologies and matches generated 16S rRNA gene sequences, a broad phylogenetic marker gene, to existing databases in order to characterize the microbial taxonomy down to the genus level [7, 8]. At the time of the 2019 guideline update, amplicon sequencing was used in the majority of wound microbiome studies. Evolving from this is metagenomics; it has the capability to identify not only microbes present in DFUs at a strain-specific level, but also their metabolic and antibiotic resistance capabilities [8, 9, 10•]. Instead of sequencing a single genetic locus (e.g., 16S rRNA), metagenomics sequences entire microbial genomes found within a sample. Because DNA is fragmented during the extraction process, sequences are computationally reassembled into full or partial genomes. Analyzing metagenomic-assembled genomes rather than a single amplicon provides both taxonomic data and information about biologic functions coded in the genome. This offers insights into what the organisms present are *capable* of doing but not what they *are* doing. Downfalls of metagenomics include financial cost, need for advanced computation, and inability to distinguish between live and dead microbes. This last shortcoming could be addressed using RNA sequencing and metatranscriptomics. Focusing on gene transcripts can yield data on metabolic activity in addition to taxonomy [11, 12].

### Concern 1: Small Sample Sizes and Selection Bias

Prior to the 2019 International Working Group on the Diabetic Foot guidelines, most molecular microbiology studies involved 20–30 patients [21–23]. One notable exception was Loesche and colleagues’ prospective cohort study of 100 patients with diabetic foot ulcers followed every 2 weeks for 26 weeks [13]. Clinical infection of the wound was not a criterion for study entry and was not reported; there was a relatively low prevalence of ischemia as measured by ankle- and toe-brachial indices. Investigators used amplicon sequencing and reported that wounds with microbiome community structures that shifted over time were more likely to heal compared to wounds with a stable microbiome community structure.

Since then, at least two additional studies enrolling 100 patients with diabetic foot ulcers have been published. Ours used the same prospective cohort as Loesche and colleagues but updated the methodology from amplicon sequencing to metagenomics [10•]. Among wounds that were able to heal by 12 weeks, we observed significant reductions in microbiome diversity immediately following sharp debridement. In particular, anaerobic bacteria were much less likely to be identified. Although the subset of patients receiving antibiotics was small, similar shifts were not observed following antibiotic administration.

Jnana and colleagues used amplicon sequencing in a cross-sectional study of 122 hospitalized patients with foot ulcers, 100 of whom had diabetes [24]. Patients were excluded if they received antibiotics in the past week. Wounds were classified using the Wagner system, and a bell-shaped distribution was seen with most patients having Wagner grade 2–4 ulcers. No specific assessment of underlying peripheral vascular disease was reported beyond the presence and extent of gangrene needed to classify a patient with a Wagner grade 4 or 5 ulcer. More bacterial diversity was seen in Wagner grade 5 ulcers compared with less severe ulcers. Although Wagner does not distinguish between dry and wet gangrene, this finding is consistent with the well-established, polymicrobial nature of wet gangrene. A counterintuitive finding was that obligate anaerobes were more abundant in Wagner grade 1 ulcers than all other ulcer severities, while facultative anaerobes were more abundant in Wagner grade 5 ulcers.

These three prospective studies are relatively large, although concerns for possible selection bias at the patient level remain legitimate. The Loesche and Kalan cohort enrolled patients with minimal vascular disease and imposed initial, antibiotic-free periods, which likely limited sampling from patients with more severe ulcers, namely wounds complicated by peripheral vascular disease and infection [10•, 13]. The Jnana study included patients across both spectrums but, due to the recruitment of hospitalized patients, likely underrepresented patients with early-stage ulcers. Neither were multicenter studies. The National Institute for Diabetes and Digestive and Kidney Disease (NIDDK) has established the Diabetic Foot Consortium, which is a promising answer to these limitations [25]. The consortium lays the foundation for a clinical trial network spanning seven academic limb salvage centers. The first volley of studies aims to identify biomarkers, such as molecular microbiology profiles, that predict wound healing.

Selection bias at the microbe level should also be considered. Traditional, culture-based methods are accepted as the gold standard for pathogen identification [6•]. However, culture-dependent methods are prone to underrepresenting the microbes present in the wound environment; fastidious microbes requiring specialized culturing procedures, such as obligate anaerobes, frequently go unidentified by traditional culture, even when they predominate in a wound environment [9, 14, 18]. The role of fungi in diabetic foot ulcers is also overlooked. Evidence suggests that some fungi are associated with delayed healing [15]. A longitudinal metagenomic study of DFU fungi using high-throughput ITS sequencing identified fungi in up to 80% of wounds (compared to 5% by culture-dependent methods run in parallel). The wound microbiome’s complex polymicrobial nature and its microenvironments, such as biofilms, further compound the issue of accurately characterizing the wound microbiome [26]. Therefore, traditional culture methods might introduce clinically relevant selection bias at the level of the microbe. Molecular sequencing may address some of the potential selection bias of traditional, culture-based studies. Typically, molecular approaches are able to identify more anaerobic organisms and those that are fastidious to culture. For instance, the Jnana study found that 22 of 100 diabetic wounds sampled were culture negative, but amplicon sequencing identified a variety of microbes in all samples [24]. Even when organisms are detected using traditional culture, molecular microbiology techniques often identify a broader array of microbes.

Moving forward, the field is shifting towards larger studies, particularly when using metagenomic sequencing. The Diabetic Foot Consortium is a promising way to address some of the concerns regarding study size and selection bias at the patient level. The Consortium’s network strives to enroll a diverse patient population in terms of demographics, comorbidities, and ulcer severities [25]. The cohort has the potential to yield more robust, generalizable findings than any preceding U.S. studies. We have the opportunity to explore whether traditional, culture-based results might be influenced by selection bias at the level of the microbe, especially if molecular microbiology techniques can hurdle the remaining concerns addressed below.

### Concern 2: Colonization Versus Infection

Distinguishing between colonization and infection has long challenged clinicians caring for patients with diabetic foot ulcers. Traditionally, we have relied upon cardinal signs of infection and weighed positive culture data from deeper structures—such as deep tissue and bone—more heavily than results from superficial swabs [6•]. This practice stems from concerns that wound surfaces are exposed to the environment and almost certainly colonized with microbes. Juxtaposing this, invasion of microbes into the deeper tissues is a characteristic of infection.

DNA-based molecular assays cannot distinguish between colonization and infection any more than traditional culture results. Both are able to identify what microbes are present in a sample, but not what they are doing. Since 2019, metatranscriptomics has emerged as a molecular approach that might provide insights into metabolic pathways associated with infection, rather than colonization. However, we acknowledge that these approaches are still under development. Existing studies demonstrate the technical feasibility of performing metatranscriptomics on diabetic foot ulcer samples. Similar to the initial genomic studies, current metatranscriptomic studies involve small sample sizes with concerns for patient-level selection bias [11, 12, 19•]. Initial results need to be confirmed in larger, more robust cohorts. However, promising lines of inquiry have been identified including tracking virulence profiles and host responses. This work lays the foundation for understanding whether there might be a common metabolic pathway—among pathogens and/or their human hosts—that indicates infection as opposed to colonization.

### Concern 3: Living Versus Dead Organisms

Amplicon sequencing provides valuable taxonomical information about microbial communities, but is unable to distinguish between living and dead organisms [11]. Metagenomic sequencing also has this drawback, although techniques to preferentially amplify DNA from live, as opposed to dead, organisms are underway [8, 9, 10•, 27]. We can distinguish live from dead organisms by investigating their transcription-level activity; metabolically active organisms are alive. RNA sequencing and metatranscriptomics can identify the microbes present, as well as their metabolic activities [11, 12]. Insights gained through these methods may be key in determining the interplay between microbes and host cells in chronic wound environments. They may also be able to disentangle pathogens from contaminants and commensals. To date, metatranscriptomic studies are small, proof-of-concept endeavors which we hope to see expanded upon soon.

### Concern 4: Antimicrobial Phenotyping

Perhaps one of the largest hurdles facing next-generation molecular assays is antimicrobial phenotyping. Amplicon sequencing, which focuses solely on a single genetic locus to identify the bacteria, provides extremely limited information regarding what antibiotic might be used in a medical context. For instance, if only *Staphylococcus aureus* is identified, a provider might forgo Gram-negative coverage. However, amplicon sequencing provides no information on whether that specific *Staphylococcus aureus* is methicillin resistant. Metagenomics improves upon this by providing some information regarding what resistance genes a bacteria may possess (e.g., mecA). Clinicians need to know whether resistance genes are turned on, or off, for more nuanced resistance mechanisms. Metatranscriptomics may fill this void. Indeed, Heravi and colleagues provided initial information on the diabetic foot infection “resistome,” or a collection of resistance genes expressed by a microbiome *in toto* [11]. Work like this needs to be expanded using larger patient cohorts and comparing transcriptomic data to conventional, culture-based antimicrobial susceptibility testing. Eventually, clinicians might benefit from comparative effectiveness trials where antibiotic regimens were selected using metagenomic/metatranscriptomic data versus standard antimicrobial susceptibility reports. Whether molecular microbiology techniques will ever supersede traditional antimicrobial susceptibility assays will depend on ensuring bioinformatic libraries are updated with emerging resistance mechanisms and data regarding new antimicrobial therapeutics. Cost-effectiveness will also be a challenge.

While it seems unlikely that next-generation molecular assays will ever fully replace culture-dependent methods, these novel techniques highlight one potentially important knowledge gap: antibiotic phenotypes within the context of the microbiome. Conventional culture-dependent methods remove a single organism from its natural environment. Information is gained about antibiotic susceptibilities in the context of that one organism. However, organisms may work in concert or depend upon one another within the microbiome [28]. Antibiotics that target a critical bacteria may result in disruption of the entire wound microbiome. This concept draws from principles of ecology and keystone species [29]. Another consideration is the presence of biofilms, which have been identified in more than 60% of chronic ulcers [26, 30]. These aggregate communities confer many advantages to microbes, including increased resilience to antimicrobials [31]. An appropriate antibiotic for treating a pathogen in isolation (as determined by conventional tests) may not be effective against pathogens embedded in a biofilm. Models that test antibiotic effectiveness on ulcer-derived biofilms, mimicking the wound environment, are under development [32]. Studying antimicrobial susceptibility within the complexities of the microbiome could yield more effective therapeutics.

### Concern 5: Correlation of Organism Abundance and Virulence Genes with Patient Outcomes

More work needs to be done to determine if organism abundance and the presence of specific virulence genes correlate with patient outcomes. Classically, clinicians have used 1 × 10^6^ organisms/mL as a threshold for distinguishing infection from colonization. However, this threshold was set with samples derived from typically sterile sites, such as bronchoalveolar lavage [33]. It is likely that wounds can harbor additional bioburden before infection is triggered. Furthermore, just because a bacteria is present in large quantities does not necessarily mean it is responsible for driving infection. Preliminary data from Radzieta and colleagues demonstrated that relatively sparse bacteria, such as *Bacillus*, can be some of the most metabolically active in diabetic foot infections [19•]. A snapshot of the taxonomic composition correlates poorly with wound severity. However, some microbiome characteristics have begun to identify biomarkers associated with patient outcomes. These biomarkers include species like Gram-positive anaerobic cocci and Bacteroides, which are associated with delayed healing or amputation, respectively [9, 34]. Specific virulence factors (like those involved in biofilm formation or toxin production), as well as the presence of virulence factors for different genera, have been correlated with poor patient outcomes or increased wound severity [9, 19•]. Again, the sample size of these studies limits the predictive power of the identified biomarkers, but, as metagenomic and metatranscriptomic studies that include patient outcome data expand, they contribute to a nuanced understanding of the microbiome and polymicrobial infections.

### Concern 6: Cost

As with all new technologies, metagenomics and metatranscriptomics are expensive. Cost is certainly a deterrent to using molecular diagnostics as part of what is already one of the most expensive complications of diabetes [35]. One avenue forward might be to use data from large metagenomics and metatranscriptomic studies to develop primer sets targeting key genes at the DNA or RNA level. The abundance of these targets could correlate with clinical infection or predict ulcer outcomes. Small, targeted primer sets are much less expensive and could then run on existing sequencing platforms or even in multiplex. Platforms are now readily available across a wide range of healthcare settings, thanks, in part, to COVID [36]. Using this existing infrastructure to implement molecular diagnostics specific to diabetic foot infections might reduce costs and help ensure equitable access to novel diagnostics.

Two basic types of molecular assays could be developed. One might be intended to screen patients with diabetic foot ulcers and identify those at high risk of limb loss who would benefit most from intense limb salvage in multidisciplinary clinics. This type of assay could be used to improve triage through the healthcare system. Such an assay would involve high patient volumes and require low costs. A second type of molecular assay might be reserved for when clinicians need a complex understanding of a specific patient’s wound microbiome and polymicrobial infection. This data might be used by specialists for relatively few patients facing advanced limb salvage situations. Such a test would involve low patient volumes but likely be more costly. The cost would be driven by the complexity of the assay, potentially a multiplexed PCR assay or selective targeted gene set derived from metagenomics and metatranscriptomic data.

### Early Insights into How the Microbiome Shifts from Harmony to Haywire Infection

Competition and cooperation between microbes in a wound environment, as well as microbe interaction with their human host, may affect the trajectory of wound healing. Not all interactions between host and microbe are detrimental to wound healing. A recent study highlighted how the presence of *Alcaligenes faecalis* improved wound healing by promoting re-epithelialization of keratinocytes [37]. Kim et al. found other bacteria (e.g., *Delftia* spp. and *Cutibacterium acnes*) associated with non-chronic or healing wounds in a diabetic mouse model [38]. Although specific modes of action for promoting healing have not been investigated, it was hypothesized that non-pathogenic colonizers of wounds may prevent colonization by pathogens. Notably, these populations were diverse and dynamic, matching previous studies indicating that more stable wound microbiomes are associated with poorer outcomes [13]. This study also found that biofilms never formed in healing wounds, even when bacteria with the ability to form biofilms were present. This may indicate that external pressures, either from the host, clinician (e.g., sharp debridement), or other microbes, prevented biofilm formation and staved off infection. Similar findings have been observed in cystic fibrosis models, where *S. aureus* prevents biofilm formation by *P. aeruginosa* [39].

However, microbial interactions can also promote infection. Interactions with some microbes can lead to the phenotypic shift of *S. aureus* to dormant, small colony variants, which reside both in DFU wound beds, and intraosseously, potentially acting as a reservoir for reinfection [40]. The wound microbiome can potentiate a usual commensal, such as *Corynebacterium striatum*, into playing a more pathogenic role [41]. Interactions have also been identified between fungal and bacterial partners in ulcers, where an interkingdom biofilm forms. Bacteria drive fungi to a more virulent phenotype [42]. These complex interactions can tip a wound into a healing or infectious state. Untangling these relationships may give insight into predicting patient outcomes.

## Conclusions

**Fig. 1:**
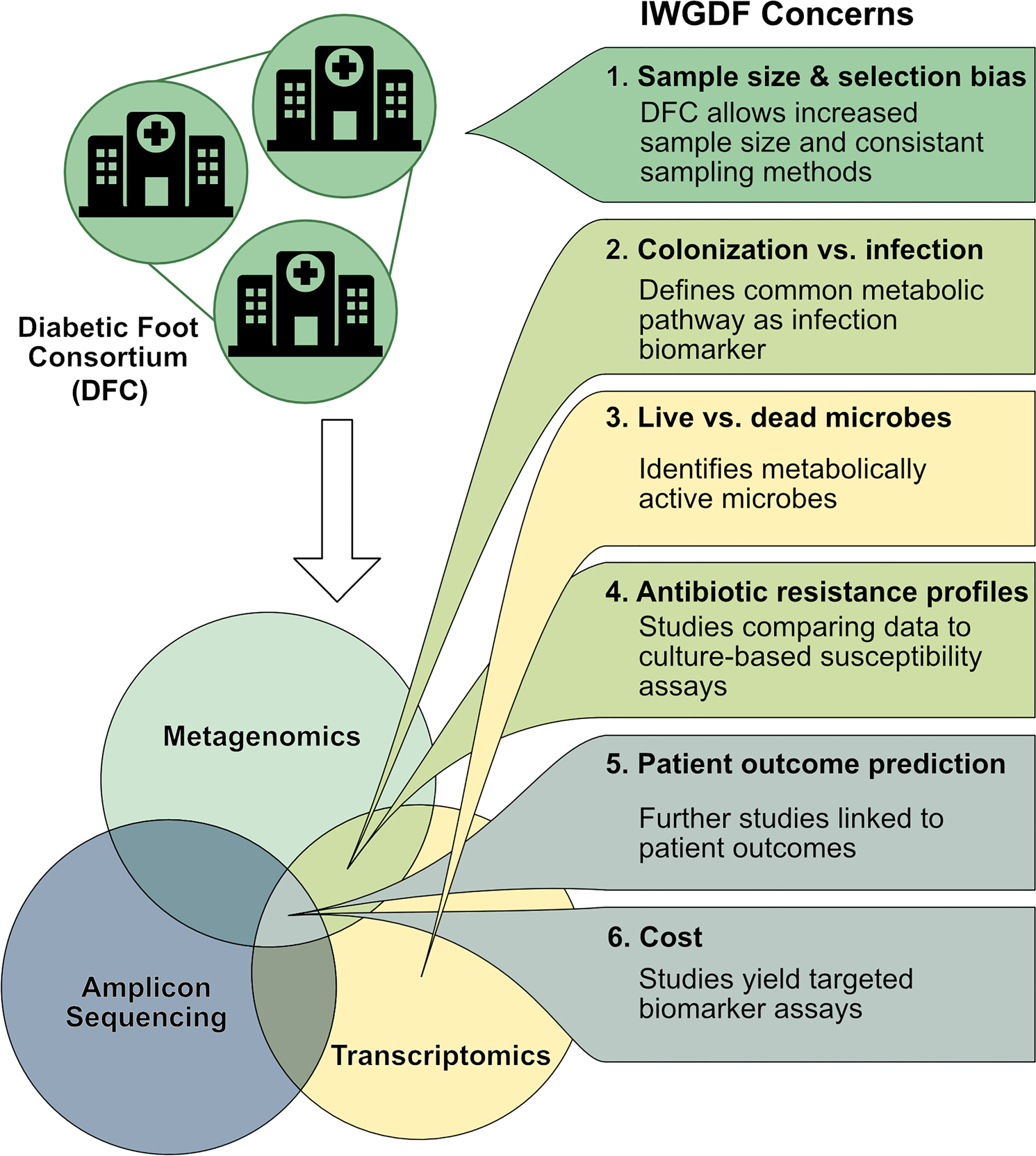
International Working Group on the Diabetic Foot’s concerns about molecular microbiology techniques and potential solutions to address them

Molecular microbiology techniques have advanced considerably since 2019. However, they are still not ready for mainstream clinical use in the diagnosis and management of infected diabetic foot ulcers. The concerns raised by the 2019 International Working Group on the Diabetic Foot guidelines are important because they: (1) invite dialogue between clinicians and molecular microbiologists and (2) highlight medically desirable attributes of a prototype assay. We hope this review summarizes advances since the guidelines were published (Fig. 1). Specifically, the Diabetic Foot Consortium offers an opportunity to address concerns around sample size and selection bias. Metagenomics and metatranscriptomics, especially used in parallel, hold promise in addressing concerns about key features of infection. Investigations that track longitudinal changes in the metagenomics and metatranscriptomic data and correlate results with clinical wound trajectories would be welcome. Additional studies comparing metatranscriptomics and culture-based antimicrobial susceptibility testing might provide high-yield information on one of the toughest hurdles facing molecular diagnostics. Finally, logistic concerns, including cost, need to be proactively addressed so that emerging diagnostics are equitably available and financially solvent. Refining our vision for how these tests will be incorporated into clinical practice is an important step along this path.

The 2019 guidelines specifically address molecular microbiology techniques in the setting of diabetic foot ulcer infections. However, it is also important to recognize that these techniques may hold prognostic value independent of their ability to diagnose infection. A molecular assay that provides information on the risk of amputation—either at the time a patient presents with an ulcer or serially as part of monitoring response to specific wound therapies—would be clinically useful. Such an assay could help triage patients into specific specialty care or trigger earlier adjustments in wound therapies to maximize limb salvage. In this regard, specifics as to infection would be desirable but not mandatory.

Finally, we need to remember that our current, culture-based methods may be generating biased information. Improving upon this might accelerate clinical advances. For instance, anaerobes and other fastidious organisms are likely underrepresented in traditional cultures but are captured using molecular approaches. Molecular ecology concepts such as keystone species might be particularly useful in considering how to treat polymicrobial infections [10•, 29]. Although exciting, these concepts remain far off from clinical applications. Understanding both the pitfalls and benefits of current and future diagnostics will help clinicians make balanced decisions when incorporating novel techniques into their daily practice. Molecular microbiology assays are still not ready for widespread clinical adoption, but ongoing collaborations between microbiologists and clinicians are the surest way to advance the field.

## Figures and Tables

**Table 1:** Differences between molecular microbiology techniques

	Amplicon sequencing	Metagenomics (whole genome sequencing)	Metatranscriptomics

**Nucleic acid analyzed**	DNA	DNA	RNA
**Sequences analyzed**	16S rRNA gene (bacteria); ITS region (fungi)	All DNA present (all taxa)	All RNA transcripts present (all taxa)
**Taxonomic resolution**	Genus (sometimes species)	Species; different clonal lineages	Species; different clonal lineages
**Distinguishes live vs. dead organisms**	No	No	Yes
**Cost**	$	$$	$$$
**Other considerations**	Requires library of 16S/ITS sequences to compare to	Technically difficult and computationally costly	Technically difficult and computationally costly
**Example studies**	Loesche et al. 2017 [13] Moon et al. 2021 [14] Kalan et al. 2016 [15] Travis et al. 2020 [16] Suryaletha et al. 2018 [17] van Asten et al. 2016 [18]	Radzieta et al. 2021 [19•] Kalan et al. 2019 [10•] Mudrik-Zohar et al. 2022 [9] Zou et al. 2020 [20]	Radzieta et al. 2021 [19•] Heravi et al. 2020 [11] Radzieta et al. 2022 [12]

*ITS* internal transcribed spacer

## Data Availability

All references for this review were accessed from PubMed.
